# RT-qPCR-Based Assessment of the Efficacy of 222 nm UVC Irradiation in Reducing SARS-CoV-2 Surface Contamination

**DOI:** 10.3390/s23136129

**Published:** 2023-07-03

**Authors:** Jurate Gruode, Arvydas Martinkenas, Mindaugas Kurmis, Darius Drungilas, Zydrunas Lukosius, Arturas Tadzijevas, Rimantas Didziokas, Valdas Jankunas, Deivydas Sapalas

**Affiliations:** Marine Research Institute, Klaipeda University, H. Manto Street 84, LT-92294 Klaipeda, Lithuania

**Keywords:** 222 nm far-ultraviolet light (UVC), SARS-CoV-2, RT-qPCR

## Abstract

Severe acute respiratory syndrome coronavirus 2 (SARS-CoV-2), which causes coronavirus disease 2019 (COVID-19), has emerged as a serious threat to human health worldwide. The effective disinfection of surfaces contaminated with SARS-CoV-2 may help prevent its spread. The aim of this study is to determine the duration required for viral RNA elimination by 222 nm far ultraviolet light using RT-qPCR as a tool. This study investigated the effect of 222 nm UVC irradiation on SARS-CoV-2 RNA in an in vitro experiment. The results showed that the copy number of SARS-CoV-2 RNA did not change even after 300 s of 222 nm UVC irradiation at 0.1 mW/cm^2^, but extending the exposure to more than 600 s reduced the number of copies of SARS-CoV-2 virus significantly. However, to fully validate the results and enhance the robustness of the findings, it is crucial to increase the number of samples analyzed in future experiments.

## 1. Introduction

The COVID-19 pandemic, caused by the SARS-CoV-2 virus, has affected society as a whole and posed major challenges, particularly in the automotive environment, where viruses are relatively common [1,2,3,4,5,6,7]. Among the various modes of transmission of infection, the World Health Organization (WHO) [8,9,10] has recognized inhaled airborne particles (those with a diameter of less than 100 µm that can remain suspended in the air) as the dominant mode of respiratory infection in the environment. Larger splash particles settle quickly due to their mass, resulting in contaminated surfaces. Sick people often touch the orofacial area unintentionally and hold on to various supports in moving transportation, increasing the likelihood of surface contamination with various viruses [7,11,12]. Public transport, in particular, with its high concentrations of people and inadequate supply of clean, germ-free air, is a place where the risk of infection is highest [12,13,14].

The experience gained since the beginning of the pandemic encourages the search for effective measures to contain the spread of the virus, in order to be prepared to contain the spread of not only this particular virus but also other possible short-lived viruses. During the COVID-19 pandemic, many countries implemented measures to reduce the spread of the virus for all modes of transmission (airborne respiratory particles, aerosolized respiratory particles, and surface contamination). The main control measures adopted were physical distancing, respiratory protection, and hand hygiene. They were designed to address the problems of aerosolized respiratory particles and virus-contaminated surfaces [15]. There is still a lack of data on the quantified risk of airborne infection in indoor environments and public transport. In fact, the first experimental evidence of SARS-CoV-2 RNA concentrations in indoor air when indoor exposure is likely [16,17,18,19], as well as traces of SARS-CoV-2 RNA in buses on surfaces, and the fact that daily commuters use public transport on buses, trolleybuses, and subways, make it clear that there is important information on the risk of infection to implement effective preventive measures, especially the use of 222 nm far ultraviolet light (UVC) for disinfection of SARS-CoV-2 surfaces. Given the number of outbreaks since the start of the pandemic, the airborne and surface transmission of SARS-CoV-2 on buses is a major concern [20,21]. Therefore, reducing the airborne transmission of respiratory pathogens on buses remains an important issue after the COVID-19 pandemic. The importance of the surface transmission of the virus has highlighted the need for adequate indoor disinfection of public transport vehicles to reduce the spread of SARS-CoV-2 [1,6,11,13]. Despite numerous studies quantifying airflow in environments, no existing standard addresses infection control requirements in non-healthcare settings [6,22]. In addition, there are no specific technical regulations or standards for buses that focus on disinfection and fresh air flows. Some countries have a standard for buses; for example, the German standard VDV 236 (2015) requires 15 m^3^/(h per person) of clean air [23].

Effective disinfection of surfaces contaminated with SARS-CoV-2 can help prevent the spread of coronavirus disease 2019 (COVID-19). Previous in vitro SARS-CoV-2 study showed that 1–3 mJ/cm^2^ of 222 nm UV irradiation (irradiance of 0.1 mW/cm^2^ for 10 and 30 s) reduced 88.5–99.7% of the SARS-CoV-2 virus detected at 50% tissue culture infectious dose (TCID 50). In contrast, the copy number of SARS-CoV-2 RNA did not change after UVC irradiation, even after 5 min of irradiation [24].

A 222 nm UV light did not damage hairless mouse skin in animal studies [25]. In a clinical study of 20 healthy people, 222 nm UV (500 mJ/cm^2^) was found to be safe and have a bactericidal effect on the human skin after 3 months of follow up [26].

The detection of viral nucleic acid via RT-qPCR is considered the gold standard viral diagnostic assay. The use of automated RT-qPCR assays for mass screening of individuals for SARS-CoV-2 has the advantage of minimal hands-on time and accuracy of results compared to conventional RT-qPCR.

The aim of this study is to determine the duration required for viral RNA elimination by 222 nm far ultraviolet light (far-UVC) using RT-qPCR as a tool. It is important for the development of methods and tools for the disinfection of SARS-CoV-2 surface contamination. 

## 2. Materials and Methods

**Figure 1:**
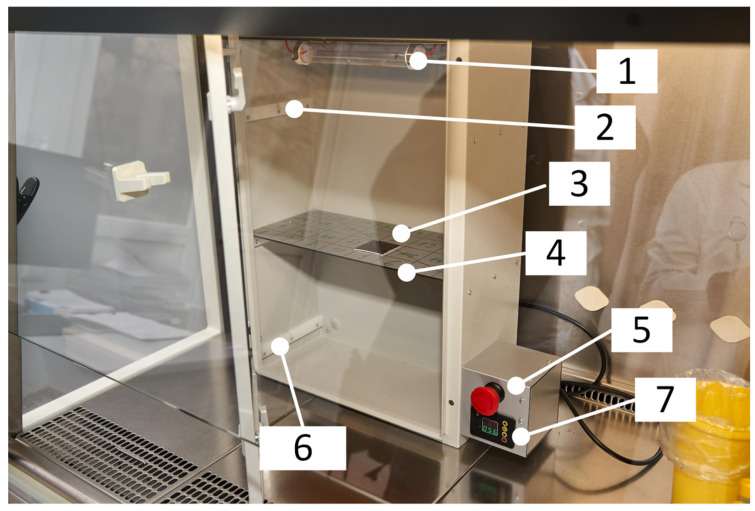
Developed 222 nm far-UVC exposition chamber (1: far-UVC bulbs; 2: Shelf I (15 cm); 3: tested sample; 4: Shelf II (30 cm); 5: emergency turn of switch; 6: Shelf III (45 cm); 7: exposure control timer.)

We used 222 nm UVC irradiation to simulate environmental cleaning. An automated UVC exposure chamber was constructed for 222 nm UVC germicidal lamp irradiation studies (Figure 1). The UVC exposure chamber is isolated from ambient light and has time setting and display functions. The ergonomic body of the apparatus, with three levels for sample placement, allows safe 222 nm irradiation tests with different samples. The amount of UVC irradiance is controlled by varying the distance of the sample from the light source: from 1.0 W/cm^2^ (bottom shelf with distance of 45 cm), 2.6 W/cm^2^ (middle shelf with distance of 30 cm), to 1.90 W/cm^2^ (top shelf with distance of 15 cm), and the radiant exposure is controlled by time.

**Figure 2:**
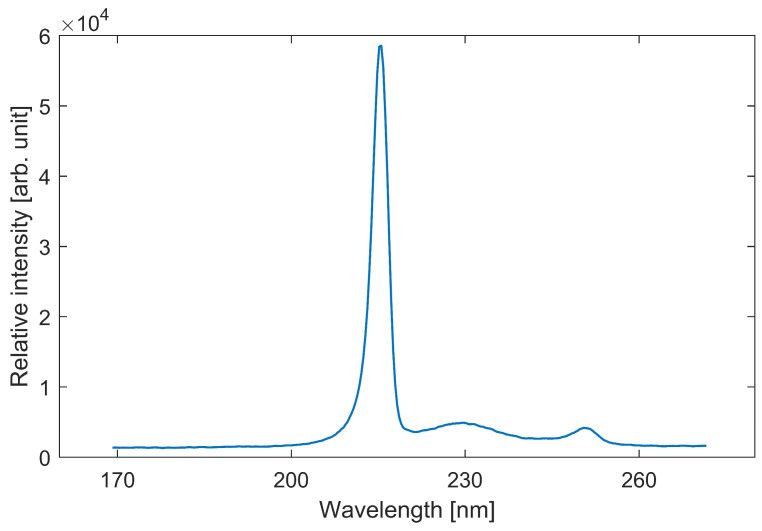
Spectrogram of the light from the used Far-UVC lamps.

The spectrum of the UVC germicidal lamps used in this study was tested using an AvaSpec ULS2048 CL spectroscope (manufacturer AVANTES B.V., the Netherlands, Apeldoorn). The spectra were measured at a distance of 5–10 cm from the light sources. The spectrograms of far-UVC disinfection and changes in the material properties of the chamber shelves were also measured with the same spectroscope. The spectrogram of direct 222 nm UVC germicidal lamp light is shown in Figure 2.

Moreover, 222 nm UVC germicidal lamps’ Far-UVC radiation intensity was measured using a Gigahertz-Optik X1-5 optometer with a UV-3727 far-UVC detector. The frequency response covers the measurement range from 200 to 300 nm. We investigated the titer of SARS-CoV-2 after UVC irradiation (from 19.0 mJ/cm^2^ to 34,220.0 mJ/cm^2^) at 222 nm for 10–1800 s (Table 1). Irradiation was varied by increasing the exposure distance from the light source and increasing the exposure time. Using the positive SARS-CoV-2 sample, the quantitative reverse transcription-polymerase chain reaction was performed to quantify SARS-CoV-2 RNA.

**Figure 3:**
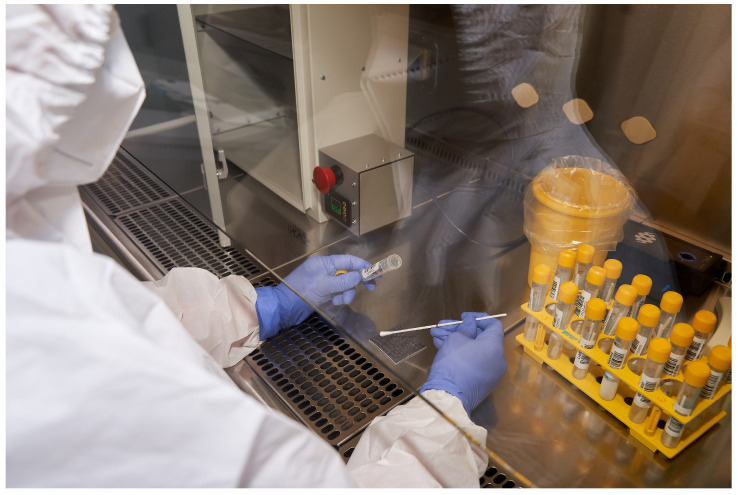
Procedure of media containing virus placement on the surface and irradiation in the developed far-UVC exposition chamber performed in the HERASAFE 2030i camera.

**Figure 4:**
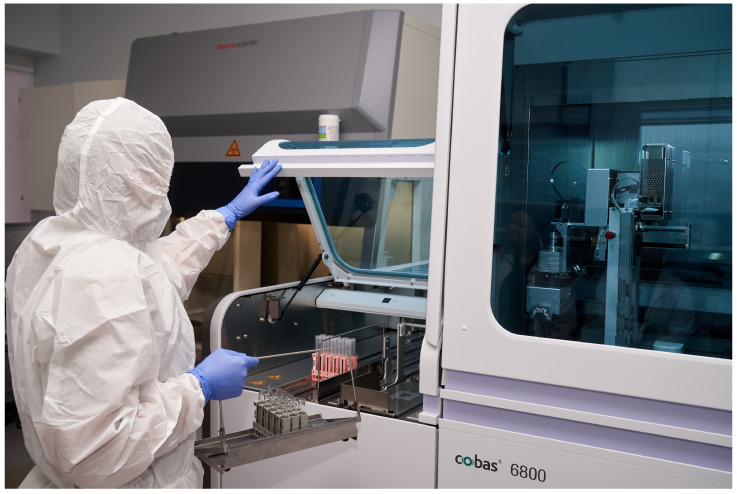
Procedure of Cobas^®^ PCR Media containing virus (positive samples) placement to the Cobas^®^ 6800 system after irradiation.

Two positive SARS-CoV-2 samples were analyzed via RT-qPCR detection at biosafety level 2 (BSL-2) on a surface simulating a public transport seat. The samples were tested before and after irradiation with modes of varying duration and distance. Figure 3 shows the HERASAFE 2030i chamber in which the automated research module was placed. Screening of SARS-CoV-2 viral RNA via reverse transcription poly-merase chain reaction (AT-qPCR) was performed using the Cobas^®^ (Indianapolis, IN, USA) 6800 system before and after 222 nm UVC irradiation at different distances and durations (Figure 4).

This study was conducted at the Molecular Diagnostics Department of Klaipeda University Hospital (a partner of Klaipeda University). The in vitro samples were applied to the plastic parts of the material commonly used in bus interiors. Sampling tampons were pulled from the surface to be tested in a zigzag pattern along the surface to be tested. Swabs were taken using fiber and polyester swabs. The sample was immediately transferred to Cobas^®^ PCR Media. Collected samples are stored at 2–8 °C and processed within 48 h.

Samples collected using the Cobas^®^ PCR Media Uni Swab Sample Kit Quantitative Reverse Transcription PCR (RT-qPCR) SARS-CoV-2 RNA was extracted from the collected viral samples, and RT-qPCR was performed using a Cobas^®^ SARS-CoV-2 Test Virus Kit according to the manufacturer’s protocol. 

Selective amplification of target nucleic acid from the sample is achieved via target-specific forward and reverse primers for the ORF1 a/b non-structural region that is unique to SARS-CoV-2. In addition, a conserved region in the structural protein envelope E-gene was selected for pan-Sarbecovirus detection. The pan-Sarbecovirus detection kits will also detect SARS-CoV-2 virus [27,28]. 

Selective amplification of the RNA internal control is achieved via non-competitive sequence-specific forward and reverse primers that have no homology to the coronavirus genome. A thermostable DNA polymerase enzyme is used for amplification. Conventional RT-qPCR for specific amplification of the ORF1 a/b non-structural region of SARS-CoV-2 and the structural protein envelope E gene was performed on the Cobas^®^ 6800 System according to the manufacturer’s protocol. A positive SARS-CoV-2 sample was used for this study, and the viral Ct values determined for the ORF1 sequences and E gene were 18 and 20, respectively [29].

In accordance with safety requirements, the Cobas^®^ PCR Media containing the positive samples was applied to PVC (polyvinyl chloride) surfaces (two 2.5 × 2.5 cm slides) using a double HEPA-filtered draft box. The first sample was not exposed to UVC radiation, while the others were exposed to radiation of varying duration and intensity depending on their distance from the lamps (Table 1). After irradiation, the residues of the transformants were collected, and the selected sequences of the SARS-CoV-2 virus RNA were measured in them according to the approved methodology.

**Figure 5:**
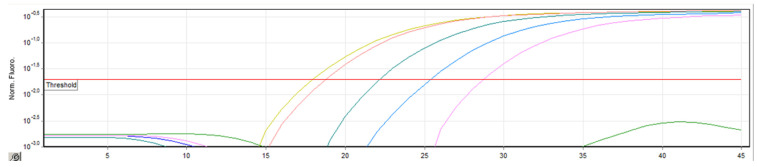
Amplification plot and threshold: visualizing fluorescence signal accumulation in real-time qPCR.

The aim of this study was to investigate the in vitro efficacy of 222 nm far-ultraviolet light (UVC) on the disinfection of SARS-CoV-2 surface contamination by measuring residual viral RNA via RT-qPCR before UV exposure and after exposure for different durations. We started the research with low average irradiation doses (mJ/cm^2^, average shelf, short times), because according to literature data 222 nm UV radiation is available for viral irradiation to inactivate viruses and phage virus surrogates, as data show that about 2 log10 reduction of coronaviruses is achieved for each 2 mJ/cm^2^ UVC dose [22,23,24,25,26]. The reduction of viral RNA can be assessed by the Ct value, which is a single data point derived from real-time qPCR amplification plots and is called the threshold cycle or “Ct”. In order to measure the reduction of viral RNA, scientists use a metric called the Ct value. This value is obtained from real-time qPCR (quantitative polymerase chain reaction) amplification plots and is commonly referred to as the “threshold cycle” or Ct. In a real-time qPCR assay, a positive reaction is identified by the accumulation of a fluorescent signal. The Ct value represents the number of cycles needed for the fluorescent signal to cross a specific threshold. Ct levels are inversely proportional to the amount of target nucleic acid in the sample (i.e., the lower the Ct level the greater the amount of target nucleic acid in the sample) [30]. The threshold in qPCR is a specific fluorescence value (ΔRn) that is chosen for an assay and used to calculate Ct values. These thresholds are established within the exponential phase of the qPCR process. On the amplification plot, which is displayed on a logarithmic scale on the *y*-axis, the exponential phases are represented by parallel lines with a positive slope (as shown in Figure 5). In the amplification plot, the threshold is depicted as a horizontal line that intersects with the amplification curve. Each sample’s fluorescence signal is plotted against the number of cycles, reflecting the progressive accumulation of the qPCR product throughout the duration of the experiment. The threshold, represented by a horizontal line, is strategically set at a specific fluorescence value within the exponential phase of the amplification. By observing the intersection or crossing of the fluorescence signal with the threshold line, the cycle threshold (Ct) values can be calculated for individual samples. This plot offers valuable insights into the amplification dynamics and helps in determining the Ct values for further analysis and interpretation.

## 3. Results

Previous studies of viral viability have shown that after 30 min of UV exposure, the viable virus is reduced by up to 90%. However, to date, the gold standard for diagnosing SARS-CoV-2 virus, both from the patient’s nasopharynx and when examining surfaces, is RT-qPCR [28]. This is why the RT-qPCR method was chosen for this study. Importantly, this method determines the presence or absence of viral RNA in the environment being tested rather than the viability of the virus itself. 

**Figure 6:**
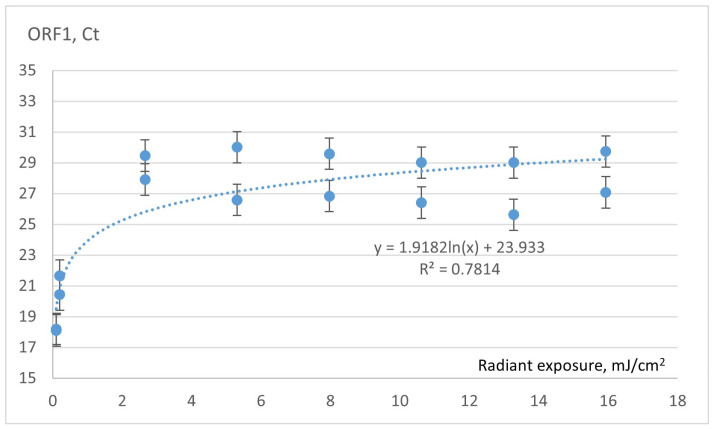
The dependence of the Ct values of the samples on UVC radiation.

Data from two separate samples positive for SARS-CoV-2 were applied to 2.5 × 2.5 cm PVC platforms and irradiated with different doses of 222 nm UVC. First, the Ct values were determined after short UVC irradiation (up to 60 s), corresponding to an exposure of up to 16 mJ/cm^2^. Figure 6 shows the dependence of the obtained Ct values on the corresponding UVC exposure up to 16 mJ/cm^2^. The data are approximated by the logarithmic curve, and the coefficient of determination is high (R^2^ = 0.78).

**Figure 7:**
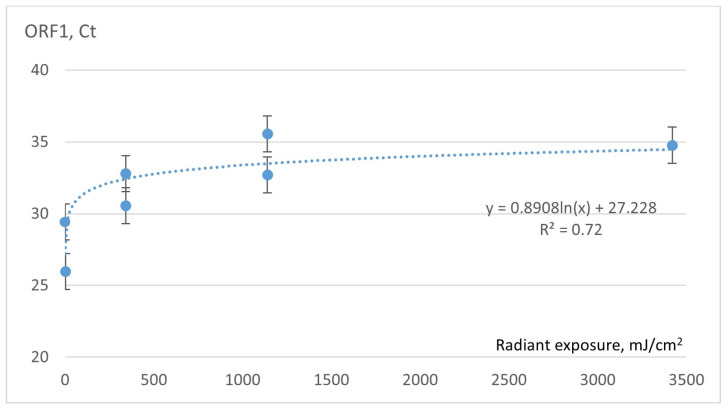
Dependence of Ct values of COVID-19 samples on intense UVC radiation.

When evaluating the short-term irradiation effect on SARS-CoV-2 samples, none of the samples showed a significant change in Ct value, indicating that no significant viral genome damage had occurred. Therefore, the long-term irradiation (up to 1800 s) was performed by increasing the irradiance even up to 3420.0 mJ/cm^2^ when the samples were placed close to the UVC lamps, on the top shelf of the UVC exposure chamber. And the evaluation of the UVC effect was carried out in three iterations: after 180 s, 600 s, and 1800 s, respectively. The results of the long-term effect of UVC irradiation on Ct values are presented in Figure 7, where the data are approximated by the logarithmic curve with the coefficient of determination (R^2^ = 0.72). In this case, the significant rise in the Ct value to nearly 35, in contrast to the Ct value obtained from the short-term irradiation, which was approximately 30, indicates a reduction of over 30 times in the copy number of SARS-CoV-2 RNA.

The change in copy number of SARS-CoV-2 RNA reduction was observed after even 180 s and 300 s of UVC irradiation, and no RNA was detected after irradiation for 1800 s. The results of changing Ct values for qPCR target regions of SARS-CoV-2 viruses before and after exposure to 222 nm UVC are shown in Table 2.

The disinfecting effect of 222 nm UVC was confirmed in this study. This study showed that the SARS-CoV-2 virus was no longer present after an average of 36.6 cycles (from 23.1 to 39.2) with 95% CI after exposure to 3420 mJ/cm^2^ for 1800 s of irradiation. In the case of sample 2, a significant increase in the Ct value is observed, but a negative value is not reached due to the higher amount of initial viral RNA. The observed gradual change in viral Ct values to complete undetectability (see the case of sample 1) after 30 min of exposure is shown in Table 3.

**Figure 8:**
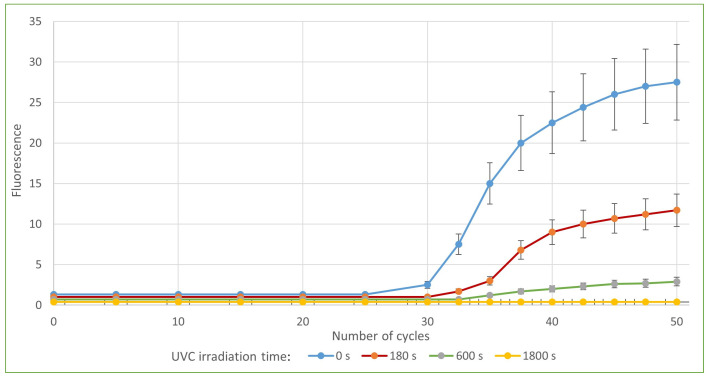
ORF1 response, by number of cycles, at different UVC irradiation durations in the upper shelf of UVC exposition chamber.

Figure 8 shows a decrease in the amount of RNA after 222 nm UVC irradiation, indicated by a later rise in the curve and a later crossing of the threshold line, resulting in a higher Ct value.

In public places (public transport, handles, handrails, and other surfaces), exposure to UVC for up to 30 s is sufficient to neutralize the viability of the virus. On the other hand, for surfaces that are important in terms of contamination (laboratories, collection points, and wards), longer exposure, up to 30 min, is recommended if the aim is not to completely neutralize the virus but to completely damage the viral RNA.

Although previous studies evaluating viral viability after exposure to 222 nm UVC radiation ranging from 2.7 mJ/cm^2^ to 15.9 mJ/cm^2^ (mean exposure time from 10 to 60 s) have found that radiation destroys viral viability, there is no evidence that short-term exposure (from 10 to 60 s) destroys viral viability. The short-term exposure (from 10 s to 60 s) is too short to degrade the viral RNA sequences, as assessed via RT-qPCR in Ct values before exposure, and after 10 s to 60 s, the amount of viral RNA did not change. However, longer durations (from 5 min to 30 min) damaged the RNA structures to minimal or undetectable levels. 

## 4. Conclusions

This study shows that 222 nm UVC irradiation is effective in reducing SARS-CoV-2 contamination. The duration of UVC exposure depends on the purpose of the tested surfaces, with short-term exposure (ranging from 2.7 mJ/cm^2^ to 15.9 mJ/cm^2^) being sufficient to destroy the viability of the virus. For complete destruction of the viral RNA and reducing it to undetectable levels, an extended UVC irradiation time of up to 30 min may be required (3420 mJ/cm^2^). To fully validate the results and enhance the robustness of the findings, it is imperative to increase the number of samples analyzed in future experiments, thus ensuring a more comprehensive evaluation of this study’s conclusions. In future works, research is needed to evaluate the safety and efficacy of this method in real-world settings and its potential to reduce the transmission of the virus. 

## Figures and Tables

**Table 1:** The radiant exposure in the far-UVC exposition chamber.

Exposure Time, s	Shelf I (Upper), mJ/cm^2^	Shelf II (Middle), mJ/cm^2^	Shelf III (Bottom), mJ/cm^2^
10	19.0	2.7	1.04
20	38.0	5.3	2.1
30	57.0	8.0	3.1
40	76.0	10.6	4.1
50	95.0	13.3	5.2
60	114.0	15.9	6.2
180	342.0	47.8	18.6
600	1140.0	159.3	62.1
1800	3420.0	477.4	186.3

**Table 2:** Results of changing Ct values for qPCR target regions of SARS-CoC-2 viruses before and after exposition 222 nm UVC.

	qPCR Target	Control	Irradiation 180 s	Irradiation 600 s	Irradiation 1800 s
Sample 1	ORF1	29.42	32.78 (11.42%)	35.57 (20.90%)	negative (100%)
	E-gene	29.95	33.72 (12.59%)	37.7 (25.88%)	negative (100%)
Sample 2	ORF1	25.97	30.56 (17.67%)	32.69 (25.88%)	34.77 (33.89%)
	E-gene	25.94	31.11 (19.93%)	32.81 (26.48%)	35.29 (36.04%)

**Table 3:** Viral Ct values at the corresponding UVC exposure.

Exposure, mJ/cm^2^	Time of UVC Exposure, s	Mean Ct Values with 95% CI (Min–Max)
0	0	29.7 (26.2–33.2)
342.0	180	33.3 (27.3–39.2)
1140.0	600	36.6 (23.1–39.2)
3420.0	1800	Not detected

## Data Availability

The data that support the findings of this study are available within the article.
